# 5-[(*E*)-(5-Bromo-2-hy­droxy­benzyl­idene)amino]-1,3,4-thia­diazole-2(3*H*)-thione

**DOI:** 10.1107/S1600536811049920

**Published:** 2011-11-25

**Authors:** Hadi Kargar, Reza Kia, Muhammad Nawaz Tahir

**Affiliations:** a Department of Chemistry, Payame Noor University, PO Box 19395-3697 Tehran, Iran; b X-ray Crystallography Laboratory, Plasma Physics Research Center, Science and Research Branch, Islamic Azad University, Tehran, Iran; c Department of Chemistry, Science and Research Branch, Islamic Azad University, Tehran, Iran; d Department of Physics, University of Sargodha, Punjab, Pakistan

## Abstract

In the title mol­ecule, C_9_H_6_BrN_3_OS_2_, the dihedral angle between the benzene ring and the five-membered ring is 5.5 (3)°. An intra­molecular O—H⋯N hydrogen bond forms an *S*(6) ring motif. In the crystal, N—H⋯S hydrogen bonds link mol­ecules into centrosymmetric dimers creating *R*_2_^2^(8) ring motifs. In addition, there are inter­molecular S⋯S [3.430 (2) Å] contacts. The crystal used was a non-merohedral twin with a ratio of 0.113 (3):0.887 (3) for the components.

## Related literature

For the biological versatility of thione ligands, see, for example: Kumar *et al.* (1988 ▶); Yadav *et al.* (1989 ▶). For related structures, see: Zhang (2003 ▶); Kargar *et al.* (2011 ▶). For hydrogen-bond motifs, see: Bernstein *et al.* (1995 ▶). For van der Waals radii, see: Bondi (1964 ▶). For standard bond lengths, see: Allen *et al.* (1987 ▶).
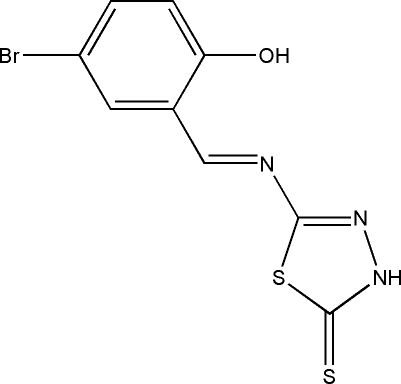


## Experimental

### 

#### Crystal data

C_9_H_6_BrN_3_OS_2_
*M* *_r_* = 316.20
Monoclinic, 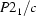

*a* = 18.3690 (13) Å
*b* = 4.0016 (3) Å
*c* = 16.2877 (13) Å
β = 112.660 (4)°
*V* = 1104.82 (14) Å^3^
*Z* = 4
Mo *K*α radiation
μ = 4.08 mm^−1^
*T* = 291 K
0.11 × 0.05 × 0.02 mm

#### Data collection

Bruker SMART APEXII CCD area-detector diffractometer
Absorption correction: multi-scan (*SADABS*; Bruker, 2005 ▶) *T*_min_ = 0.663, *T*_max_ = 0.923
10313 measured reflections
2734 independent reflections
1760 reflections with *I* > 2σ(*I*)
*R*_int_ = 0.071

#### Refinement

*R*[*F* ^2^ > 2σ(*F* ^2^)] = 0.060
*wR*(*F* ^2^) = 0.140
*S* = 1.08
2734 reflections
147 parameters
H-atom parameters constrained
Δρ_max_ = 1.25 e Å^−3^
Δρ_min_ = −0.71 e Å^−3^

### 

Data collection: *APEX2* (Bruker, 2005 ▶); cell refinement: *SAINT* (Bruker, 2005 ▶); data reduction: *SAINT*; program(s) used to solve structure: *SHELXTL* (Sheldrick, 2008 ▶); program(s) used to refine structure: *SHELXTL*; molecular graphics: *SHELXTL*; software used to prepare material for publication: *SHELXTL* and *PLATON* (Spek, 2009 ▶).

## Supplementary Material

Crystal structure: contains datablock(s) global, I. DOI: 10.1107/S1600536811049920/lh5379sup1.cif

Structure factors: contains datablock(s) I. DOI: 10.1107/S1600536811049920/lh5379Isup2.hkl

Supplementary material file. DOI: 10.1107/S1600536811049920/lh5379Isup3.cml

Additional supplementary materials:  crystallographic information; 3D view; checkCIF report

## Figures and Tables

**Table 1:** Hydrogen-bond geometry (Å, °)

*D*—H⋯*A*	*D*—H	H⋯*A*	*D*⋯*A*	*D*—H⋯*A*
O1—H1⋯N1	0.82	1.94	2.664 (7)	147
N3—H3⋯S2^i^	0.97	2.36	3.327 (5)	173

Symmetry code: (i) 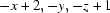.

**Fig. 1.**
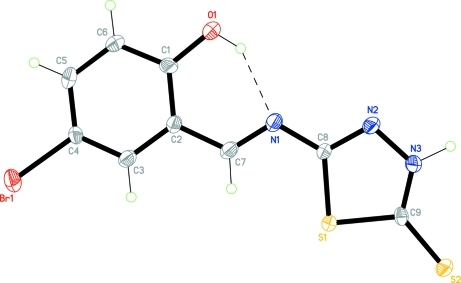
The molecular structure of the title compound, showing 40% probability displacement ellipsoids. The dashed line shows an intramolecular hydrogen bond.

**Fig. 2.**
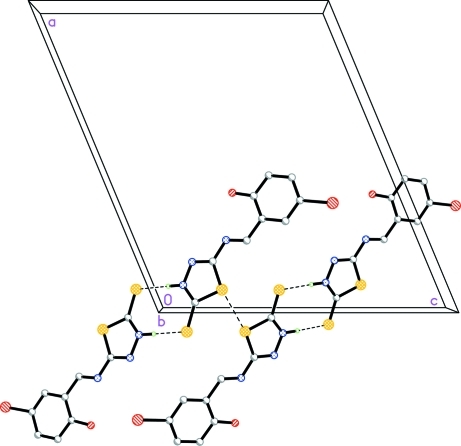
A packing diagram of the title compound viewed along the b-axis showing short intermolecular S···S contacts and centrosymmetric dimers formed by intermolecular N—H···S hydrogen bonds. The dashed lines indicate the intermolecular interactions.

## supplementary crystallographic information

### Comment

The biological versatility of compounds incorporating a thiadiazole ring is well known (Kumar *et al.*, 1988; Yadav *et al.*, 1989). In continuation of our work on the crystal structure of thione-Schiff base ligands (Kargar *et al.,* 2011), we have determined the crystal structure of the title compound.

The asymmetric unit of the title compound is shown  in Fig. 1. The bond lengths (Allen *et al.,* 1987) and angles are within the normal ranges and are comparable to related structures (Kargar *et al.,* 2011; Zhang, 2003).

The dihedral angle between the benzene ring and the five-membered ring is 5.5 (3)°. An intramolecular O—H···N hydrogen bond forms an S(6) ring motif. Intermolecular N—H···S interactions link molecules into centrosymmetric dimers with R_2_^2^(8) ring motifs (Bernstein *et al.*,1995). An interesting feature of the crystal structure is the short S1···S1^i^ [3.430 (2)Å; (i) 2 - x, -1/2 + y, 1/2 - z] contact which is shorter than the sum of the Van der Waals radius of S [3.60Å] atoms (Bondi, 1964). The crystal was a non-merohedral twin with a refined BASF ratio of 0.113 (3)/0.887 (3).

### Experimental

The title compound was synthesized by adding 5-bromo-salicylaldehyde (1 mmol) to a solution of 5-aminothiophene-2-thiol (1 mmol) in ethanol (30 ml). The mixture was refluxed with stirring for half an hour. The resultant solution was filtered. Yellow single crystals of the title compound suitable for *X*-ray structure determination were recrystallized from ethanol by slow evaporation of the solvent at room temperature over several days.

### Refinement

All hydrogen atoms were positioned geometrically with C—H = 0.93 Å, N—H = 0.97 Å, O—H = 0.82 Å and included in a riding-model approximation with U_iso_(H) = 1.2U_eq_(C,N) and 1.5 U_eq_(O). The crystal was a non-merohedral twin {Twin Law (1 0 0)[5 0 2]} which was treated TwinRotMat routine in PLATON (Spek, 2009) with a refined BASF ratio of 0.113 (3)/0.887 (3).

### Crystal data

**Table:**

C_9_H_6_BrN_3_OS_2_	*F*(000) = 624
*M**_r_* = 316.20	*D*_x_ = 1.901 Mg m^−^^3^
Monoclinic, *P*2_1_/*c*	Mo *K*α radiation, λ = 0.71073 Å
Hall symbol: -P 2ybc	Cell parameters from 2573 reflections
*a* = 18.3690 (13) Å	θ = 2.5–29.3°
*b* = 4.0016 (3) Å	µ = 4.08 mm^−^^1^
*c* = 16.2877 (13) Å	*T* = 291 K
β = 112.660 (4)°	Block, yellow
*V* = 1104.82 (14) Å^3^	0.11 × 0.05 × 0.02 mm
*Z* = 4	

### Data collection

**Table:**

Bruker SMART APEXII CCD area-detector diffractometer	10313 independent reflections
Radiation source: fine-focus sealed tube	1760 reflections with *I* > 2σ(*I*)
graphite	*R*_int_ = 0.071
φ and ω scans	θ_max_ = 28.3°, θ_min_ = 1.2°
Absorption correction: multi-scan (*SADABS*; Bruker, 2005)	*h* = −24→24
*T*_min_ = 0.663, *T*_max_ = 0.923	*k* = −5→5
2734 measured reflections	*l* = −21→21

### Refinement

**Table:**

Refinement on *F*^2^	Primary atom site location: structure-invariant direct methods
Least-squares matrix: full	Secondary atom site location: difference Fourier map
*R*[*F*^2^ > 2σ(*F*^2^)] = 0.060	Hydrogen site location: inferred from neighbouring sites
*wR*(*F*^2^) = 0.140	H-atom parameters constrained
*S* = 1.08	*w* = 1/[σ^2^(*F*_o_^2^) + (0.0412*P*)^2^ + 3.7473*P*] where *P* = (*F*_o_^2^ + 2*F*_c_^2^)/3
2734 reflections	(Δ/σ)_max_ < 0.001
147 parameters	Δρ_max_ = 1.25 e Å^−^^3^
0 restraints	Δρ_min_ = −0.71 e Å^−^^3^

### Special details

**Table:**

Geometry. All esds (except the esd in the dihedral angle between two l.s. planes) are estimated using the full covariance matrix. The cell esds are taken into account individually in the estimation of esds in distances, angles and torsion angles; correlations between esds in cell parameters are only used when they are defined by crystal symmetry. An approximate (isotropic) treatment of cell esds is used for estimating esds involving l.s. planes.
Refinement. Refinement of F^2^ against ALL reflections. The weighted R-factor wR and goodness of fit S are based on F^2^, conventional R-factors R are based on F, with F set to zero for negative F^2^. The threshold expression of F^2^ > 2sigma(F^2^) is used only for calculating R-factors(gt) etc. and is not relevant to the choice of reflections for refinement. R-factors based on F^2^ are statistically about twice as large as those based on F, and R- factors based on ALL data will be even larger.

### Fractional atomic coordinates and isotropic or equivalent isotropic displacement parameters (Å^2^)

**Table:**

	*x*	*y*	*z*	*U*_iso_*/*U*_eq_	
Br1	0.65016 (4)	0.82937 (19)	−0.22477 (5)	0.0438 (2)	
S1	0.92727 (8)	0.4125 (4)	0.25824 (11)	0.0306 (4)	
S2	1.06396 (9)	0.3421 (4)	0.43539 (10)	0.0329 (4)	
O1	0.6140 (2)	0.1515 (13)	0.0870 (3)	0.0439 (12)	
H1	0.6563	0.1408	0.1296	0.066*	
N1	0.7683 (3)	0.2398 (13)	0.1762 (3)	0.0314 (12)	
N2	0.8404 (3)	0.0884 (14)	0.3226 (4)	0.0355 (13)	
N3	0.9159 (3)	0.1150 (13)	0.3865 (3)	0.0347 (13)	
H3	0.9211	−0.0002	0.4408	0.042*	
C1	0.6244 (3)	0.3022 (18)	0.0192 (4)	0.0340 (15)	
C2	0.6990 (3)	0.4180 (15)	0.0246 (4)	0.0286 (13)	
C3	0.7065 (3)	0.5752 (15)	−0.0484 (4)	0.0315 (14)	
H3A	0.7556	0.6477	−0.0453	0.038*	
C4	0.6402 (3)	0.6216 (15)	−0.1255 (4)	0.0313 (14)	
C5	0.5670 (4)	0.5144 (17)	−0.1308 (4)	0.0365 (15)	
H5A	0.5229	0.5488	−0.1829	0.044*	
C6	0.5587 (3)	0.3570 (17)	−0.0598 (4)	0.0367 (15)	
H6A	0.5091	0.2864	−0.0642	0.044*	
C7	0.7688 (3)	0.3839 (16)	0.1055 (4)	0.0339 (15)	
H7A	0.8162	0.4697	0.1066	0.041*	
C8	0.8377 (3)	0.2352 (15)	0.2507 (4)	0.0260 (13)	
C9	0.9707 (3)	0.2737 (14)	0.3678 (4)	0.0261 (13)	

### Atomic displacement parameters (Å^2^)

**Table:**

	*U*^11^	*U*^22^	*U*^33^	*U*^12^	*U*^13^	*U*^23^
Br1	0.0511 (4)	0.0493 (4)	0.0304 (4)	0.0088 (3)	0.0152 (3)	0.0084 (3)
S1	0.0242 (7)	0.0387 (9)	0.0263 (8)	−0.0014 (6)	0.0067 (6)	0.0065 (7)
S2	0.0252 (7)	0.0429 (9)	0.0264 (9)	−0.0014 (7)	0.0055 (6)	−0.0007 (7)
O1	0.030 (2)	0.065 (3)	0.033 (3)	−0.009 (2)	0.008 (2)	0.008 (3)
N1	0.026 (3)	0.039 (3)	0.026 (3)	0.002 (2)	0.006 (2)	0.001 (2)
N2	0.024 (2)	0.051 (3)	0.029 (3)	−0.006 (2)	0.006 (2)	0.003 (3)
N3	0.033 (3)	0.047 (3)	0.024 (3)	−0.005 (2)	0.010 (2)	0.006 (3)
C1	0.029 (3)	0.053 (4)	0.024 (3)	−0.008 (3)	0.014 (3)	−0.004 (3)
C2	0.027 (3)	0.031 (3)	0.026 (3)	0.001 (2)	0.008 (3)	0.001 (3)
C3	0.024 (3)	0.041 (4)	0.028 (3)	0.002 (3)	0.008 (3)	0.000 (3)
C4	0.033 (3)	0.032 (3)	0.026 (3)	0.001 (3)	0.007 (3)	0.003 (3)
C5	0.033 (3)	0.044 (4)	0.025 (3)	0.003 (3)	0.003 (3)	−0.002 (3)
C6	0.024 (3)	0.045 (4)	0.037 (4)	−0.003 (3)	0.008 (3)	0.001 (3)
C7	0.023 (3)	0.042 (4)	0.034 (4)	0.002 (3)	0.007 (3)	−0.001 (3)
C8	0.021 (3)	0.033 (3)	0.023 (3)	0.003 (2)	0.007 (2)	0.003 (2)
C9	0.032 (3)	0.026 (3)	0.021 (3)	0.002 (2)	0.010 (3)	−0.003 (2)

### Geometric parameters (Å, °)

**Table:**

Br1—C4	1.889 (6)	C1—C6	1.402 (8)
S1—C9	1.741 (6)	C1—C2	1.417 (8)
S1—C8	1.752 (6)	C2—C3	1.399 (8)
S2—C9	1.663 (6)	C2—C7	1.448 (8)
O1—C1	1.334 (7)	C3—C4	1.384 (8)
O1—H1	0.8200	C3—H3A	0.9300
N1—C7	1.292 (8)	C4—C5	1.382 (9)
N1—C8	1.381 (7)	C5—C6	1.377 (9)
N2—C8	1.293 (8)	C5—H5A	0.9300
N2—N3	1.381 (7)	C6—H6A	0.9300
N3—C9	1.322 (8)	C7—H7A	0.9300
N3—H3	0.9690		
			
C9—S1—C8	89.4 (3)	C5—C4—Br1	119.7 (5)
C1—O1—H1	109.5	C3—C4—Br1	119.6 (5)
C7—N1—C8	117.9 (5)	C6—C5—C4	120.6 (6)
C8—N2—N3	108.9 (5)	C6—C5—H5A	119.7
C9—N3—N2	119.8 (5)	C4—C5—H5A	119.7
C9—N3—H3	128.5	C5—C6—C1	120.6 (6)
N2—N3—H3	111.7	C5—C6—H6A	119.7
O1—C1—C6	118.9 (5)	C1—C6—H6A	119.7
O1—C1—C2	122.7 (6)	N1—C7—C2	123.0 (6)
C6—C1—C2	118.4 (6)	N1—C7—H7A	118.5
C3—C2—C1	120.3 (6)	C2—C7—H7A	118.5
C3—C2—C7	118.2 (5)	N2—C8—N1	120.1 (5)
C1—C2—C7	121.5 (6)	N2—C8—S1	114.4 (4)
C4—C3—C2	119.4 (5)	N1—C8—S1	125.5 (5)
C4—C3—H3A	120.3	N3—C9—S2	127.4 (5)
C2—C3—H3A	120.3	N3—C9—S1	107.5 (4)
C5—C4—C3	120.8 (6)	S2—C9—S1	125.1 (4)
			
C8—N2—N3—C9	0.2 (8)	C8—N1—C7—C2	−177.1 (5)
O1—C1—C2—C3	−179.9 (6)	C3—C2—C7—N1	−179.2 (6)
C6—C1—C2—C3	−1.7 (10)	C1—C2—C7—N1	1.9 (10)
O1—C1—C2—C7	−1.1 (10)	N3—N2—C8—N1	179.3 (5)
C6—C1—C2—C7	177.1 (6)	N3—N2—C8—S1	−0.1 (7)
C1—C2—C3—C4	1.1 (9)	C7—N1—C8—N2	−178.7 (6)
C7—C2—C3—C4	−177.7 (6)	C7—N1—C8—S1	0.6 (8)
C2—C3—C4—C5	−0.1 (9)	C9—S1—C8—N2	0.0 (5)
C2—C3—C4—Br1	−179.3 (5)	C9—S1—C8—N1	−179.3 (5)
C3—C4—C5—C6	−0.4 (10)	N2—N3—C9—S2	178.3 (5)
Br1—C4—C5—C6	178.8 (5)	N2—N3—C9—S1	−0.2 (7)
C4—C5—C6—C1	−0.1 (10)	C8—S1—C9—N3	0.1 (5)
O1—C1—C6—C5	179.4 (6)	C8—S1—C9—S2	−178.4 (4)
C2—C1—C6—C5	1.2 (10)		

### Hydrogen-bond geometry (Å, °)

**Table:**

*D*—H···*A*	*D*—H	H···*A*	*D*···*A*	*D*—H···*A*
O1—H1···N1	0.82	1.94	2.664 (7)	147
N3—H3···S2^i^	0.97	2.36	3.327 (5)	173

Symmetry codes: (i) −*x*+2, −*y*, −*z*+1.
